# Incidence, mortality, and survival analyses of patients with thymic lymphoma

**DOI:** 10.3389/fonc.2022.933672

**Published:** 2022-09-14

**Authors:** Li Wang, Zhile Wang, Lanqing Huo, Ailin Zhao

**Affiliations:** ^1^ Department of Hematology, West China Hospital, Sichuan University, Chengdu, China; ^2^ Department of Thoracic Surgery, West China Hospital, Sichuan University, Chengdu, China; ^3^ Department of Radiation Oncology, Sun Yat-sen University Cancer Center, State Key Laboratory of Oncology in South China, Guangzhou, China

**Keywords:** prognosis, radiotherapy, thymic lymphoma, surgery, chemotherapy

## Abstract

**Objectives:**

To explore the clinical and prognostic characteristics of thymic lymphoma and the effects of current treatments on the prognosis.

**Methods:**

Patients diagnosed as primary thymic lymphoma between 1975 and 2018 from the nine states of the US were identified, including Atlanta, Connecticut, Detroit, Hawaii, Iowa, New Mexico, San Francisco-Oakland, Seattle–Puget Sound, and Utah. Incidence and mortality rates were analyzed using SEER*Stat 8.3.9 software. Univariate and multivariate Cox regressions were performed to identify prognostic factors. The Kaplan–Meier curve and log-rank test were used to compare overall survival (OS) among different treatments.

**Results:**

A total of 233 patients diagnosed as thymic lymphoma were identified, and eight of them were lost to follow-up or died upon diagnosis. The incidence of thymic lymphoma was 2.032 per ten million (95% CI: 1.777–2.312), and the mortality rate was 0.649 per ten million (95% CI: 0.508–0.817). Among the 225 patients with definite follow-up, 98 were males and 127 were females, with a median age of 33 years. The Cox regression results showed that age and pathological type were independent risk prognostic factors. The 5-, 10-, and 20-year OS were 80.0%, 77.5%, and 70.9%, respectively. For Ann Arbor stage I and II patients, there was no significant difference between the surgical group (N = 78) and the non-operative group (N = 65; P = 0.270). The radiotherapy group (N = 79) had better OS than the non-radiotherapy group (N = 64) in the first 25 years, and the prognosis in the later years was not significantly different (P = 0.051). The chemotherapy group (N = 37) had a significantly better prognosis than the non-chemotherapy group (N = 37; P = 0.020). Patients who received postoperative radiotherapy (N = 45) or who only received radiotherapy (N = 34) seemed to have better OS than that of patients who only received surgery (N = 33), although the difference was not significant (P = 0.063).

**Conclusions:**

Age and pathological type were independent prognostic factors for thymic lymphoma. Surgical treatment had limited effects on OS, while both radiotherapy and chemotherapy could significantly improve the survival outcome.

## Introduction

Lymphoma is one of the most common malignancies which can occur in different organs and tissues throughout the body, which includes Hodgkin’s lymphoma (HL) and non-Hodgkin’s lymphoma (NHL) (1). Thymic lymphoma is a rare kind of tumor growing in the thymus (2) and is difficult to distinguish from thymoma by images. NHL in the thymus includes diffuse large B-cell lymphoma (DLBCL) and T-lymphoblastic lymphoma originated primarily in the mediastinum. Among them, mucosa-associated lymphoid tissue (MALT) lymphoma in the thymus was rarely found and was firstly reported in 1990 by Isaacson et al. (3). Currently, although some conventional treatments including surgery, radiotherapy, and chemotherapy have been applied to thymic lymphoma treatment, there is still no standard treatment. According to the WHO classification of lymphoma, primary thymic large B-cell lymphoma (PTLBL) is the predominant pathological type of thymus lymphoma (4). Based on previous studies, there were only case reports or cases series of small sample size for thymic lymphoma (5–10). Therefore, clinical data and evidence for thymic lymphoma are lacking, and a larger cohort of the disease is highly required.

At present, surgery is thought to be one of the critical managements for thymic lymphoma, but the resection criteria are obscure. Besides, whether patients can obtain survival benefits from postoperative treatments including radiotherapy and chemotherapy remains debated. Thus, we performed multiple clinical analyses using the SEER (Surveillance, Epidemiology, and End Results) database, the largest clinical cancer database in the world, which contains clinical data about one-third of cancer patients in the US. Since thymic lymphoma occurs quite rarely, few previous studies reported its incidence and mortality. Besides, there is still lack of survival data of the disease. Thus, from the SEER database, we collected and analyzed patients diagnosed as thymic lymphoma from 1975 to 2018 in the US. To our knowledge, this is the largest cohort of thymic lymphoma. Not only were incidence and mortality reported but also survival outcome was analyzed for treatment comparisons and prognostic factor identification.

## Methods

### Data source

Using SEER*Stat 8.3.9 software (https://seer.cancer.gov/seerstat), patients’ data from the SEER Program of the National Cancer Institute across nine cancer registries in the United States were used for the present study, including Atlanta, Connecticut, Detroit, Hawaii, Iowa, New Mexico, San Francisco-Oakland, Seattle–Puget Sound, and Utah. Primary cancer site and histology were coded using the third edition of the International Classification of Diseases for Oncology (ICD-O-3).

### Study population

Patients diagnosed as thymic lymphoma between 1975 and 2018 were identified from the SEER database. The search strategy was set as “Site Recode B ICD-O-3/WHO 2008 = Lymphoma AND Primary Site = Thymus,” which indicated that the inclusion criteria included patients diagnosed as primary thymic lymphoma confirmed by histology. A total of 233 patients were retrieved in the SEER database. Of them, six patients were dead at diagnosis and two patients had no definite time of survival. Thus, 225 patients were finally included for clinical prognostic analysis. All patient information in the SEER database was anonymous, and informed consent was waived by the Ethics Committee of West China Hospital.

### Study variables and survival data

Clinical and pathological characteristics were directly collected from the SEER database including age, sex, race, time of diagnosis, lymphoma subtype, histological type, Ann Arbor stage, surgery, radiotherapy, chemotherapy, and cause of death. Survival data were recorded since the diagnosis of thymic lymphoma. Ethnicity was divided into white, black, and others (American Indian/Alaska Native, Asian, or Pacific Islander). The histological types included PTLBL, nodular sclerosis classical Hodgkin lymphoma (NSCHL), DLBCL, and other sporadic types.

### Statistical analysis

Using SEER Stat software, incidence and mortality rates among patients with thymic lymphoma from 1975 to 2018 were estimated. The SPSS statistical software (version 25.0) was used to perform univariate and multivariate Cox regression analyses, and the hazards ratio (HR) and 95% confidence interval (CI) were calculated. R software’s (version 4.1.1; http://www.r-project.org) “SurvMiner package” and “Survival package” were used to describe the Kaplan–Meier survival curve and survival information of different groups. Furthermore, the log-rank test was used to compare overall survival (OS). All statistical tests were conducted by two-sided tests, and P < 0.05 was considered statistically significant.

## Results

### Incidence and mortality

**Figure 1:**
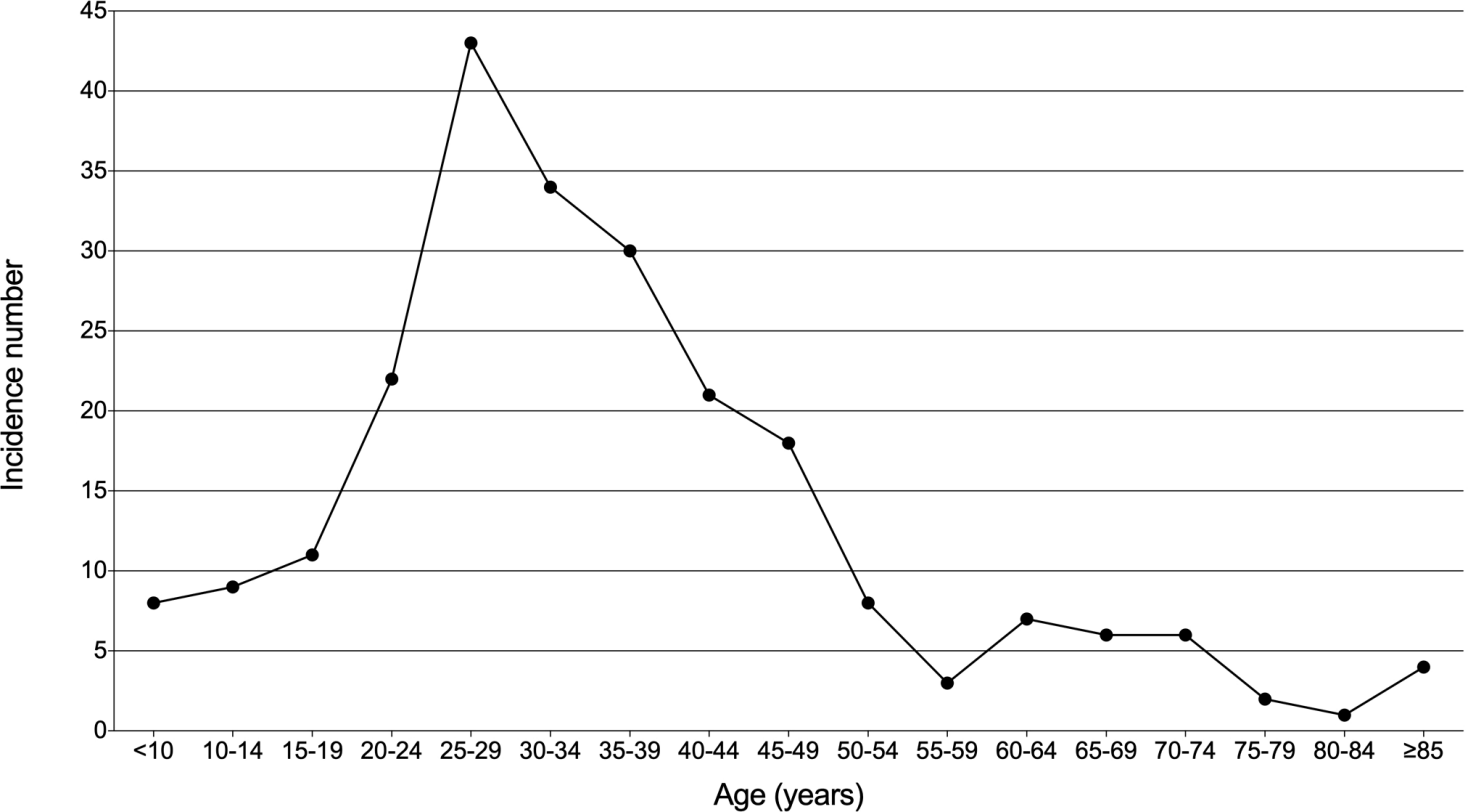
Age distribution of incidence in patients with thymic lymphoma from the SEER Research Data with 9 Registries (1975–2018).

Morbidity and mortality rates were calculated for patients with thymic lymphoma in the SEER database. From 1975 to 2018, the total observed population was 1,123,541,948 in this database. The incidence and mortality of thymic lymphoma were 2.032 (95% CI: 1.777–2.312) and 0.649 (95% CI: 0.508–0.817) per ten million persons, respectively. In **Figure 1**, age distribution of its incidence was shown. Thymic lymphoma was enriched in young and middle-aged adults and was relatively rare among those more than 50 years old.

### Demographic characteristics

A total of 255 patients with thymic lymphoma were included in the clinical outcome analysis (**Table 1**), including 98 (43.6%) men and 127 (56.4%) women with a median age of 33 years (interquartile range, IQR: 26–44). Most of them were white (173, 76.9%); others included black (30, 13.3%), other races (18, 8.0%), and unknown races (4, 1.8%). Fifty-six cases (24.9%) were diagnosed from 1975 to 1990, 45 cases (20.0%) from 1991 to 2000, 68 cases (30.2%) from 2001 to 2010, and 56 cases (24.9%) from 2011 to 2018. Sixty-three patients (28.0%) were HL, and 162 patients (72.0%) were NHL. As for histological types, 63 (28.0%) of them were PTLBL, 58 (25.8%) were NSCHL, 54 (24.0%) were DLBCL, and 50 (22.2%) were other sporadic types. Among them, 72 (32.0%) patients were recorded as Ann Arbor stage I, 71 (31.6%) as Ann Arbor stage II, six (2.4%) as Ann Arbor stage III, 23 (9.0%) as Ann Arbor stage IV, and 53 (23.6%) as unknown stage. There were 124 patients (55.1%) who underwent surgery, while 111 (49.3%) patients received radiotherapy and 159 (70.7%) received chemotherapy.

**Table 1:** Univariate Cox regression analyses for overall survival in 225 patients with thymic lymphoma.

Characteristics	N = 225 (%)	Univariate analysis			Multivariate analysis		
		HR	95% CI	P value	HR	95% CI	P value
Age, years	33 (26-44)	1.04	1.02-1.05	<0.001	1.03	1.03-1.04	<0.001
Sex							
Male	98 (43.6)	Referencee	–	–	Reference	–	–
Female	127 (56.4)	0.56	0.34-0.91	0.019	0.66	0.38-1.13	0.131
Race							
White	173 (76.9)	Reference	–	–			
Black	30 (13.3)	1.23	0.60-2.51	0.576			
Others	18 (8.0)	0.68	0.21-2.18	0.513			
Unknown	4 (1.8)	–	–	–			
Diagnosis year							
1975-1990	56 (24.9)	Reference	–	–	Reference	–	–
1991-2000	45 (20.0)	0.8	0.43-1.49	0.484	0.5	0.25-0.98	0.043
2001-2010	68 (30.2)	0.48	0.23-0.96	0.039	0.38	0.15-0.89	0.026
2011-2018	56 (24.9)	0.21	0.06-0.70	0.011	0.21	0.05-0.86	0.029
Lymphoma type							
HL	63 (28.0)	Reference	–	–	Reference	–	–
NHL	162 (72.0)	1.75	1.01-3.03	0.047	1.96	0.63-6.15	0.247
Pathologic type							
PTLBL	63 (28.0)	Reference	–	–	Reference	–	–
NSCHL	58 (25.8)	2.82	0.81-9.83	0.104	2.4	0.42-13.85	0.328
DLBCL	54 (24.0)	4.63	1.34-16.02	0.016	2.78	0.71-10.90	0.143
Others	50 (22.2)	10.9	3.32-36.23	<0.001	4.71	1.16-19.22	0.031
Ann Arbor stage							
I	72 (32.0)	Reference	–	–			
II	71 (31.6)	1.25	0.67-2.35	0.486			
III+IV	29 (12.9)	1.04	0.41-2.61	0.938			
Unknown	53 (23.6)	1.67	0.88-3.18	0.115			
Surgery							
Yes	101 (44.9)	1.84	0.99-3.42	0.054	0.96	0.42-2.17	0.917
No	124 (55.1)	Reference	–	–	Reference	–	–
Radiotherapy							
Yes	111 (49.3)	Reference	–	–			
No	114 (50.7)	1.17	0.72-1.90	0.52			
Chemotherapy							
Yes	159 (70.7)	0.71	0.43-1.20	0.201			
No	66 (29.3)	Reference	–	–			

HL, Hodgkin lymphoma; NHL, non-Hodgkin lymphoma; PTLBL, primary thymic large B-cell lymphoma; NSCHL, nodular sclerosis classical Hodgkin lymphoma; DLBCL, diffuse Large B-cell lymphoma.

### Univariate and multivariate Cox regression analyses

Univariate and multivariate Cox regression analyses were performed for 255 thymic lymphoma patients to identify their prognostic factors. Univariate analysis showed that age (HR = 1.04, 95% CI: 1.02–1.05, P < 0.001), gender (female vs. male, HR = 0.56, 95% CI: 0.34–0.91, P = 0.019), time of diagnosis (1975–1990 as reference: 2001–2010, HR = 0.48, 95% CI: 0.23–0.96, P = 0.039; 2011–2018, HR = 0.21, 95% CI: 0.06–0.70, P = 0.011), lymphoma types (NHL vs. HL: HR = 1.75, 95% CI: 1.01–3.03, P = 0.047), and pathological types (PTLBL as reference: DLBCL, HR = 4.63, 95% CI: 1.34–16.02, P = 0.016; other types, HR = 10.9, 95% CI: 3.32–36.23, P < 0.001) were significantly associated with OS. According to univariate analysis, variables with P < 0.10 were included in multivariate Cox regression analysis, and the results found that higher age (HR = 1.03, 95% CI: 1.03–1.04, P < 0.001), earlier diagnosis (1975–1990 as reference: 1991–2000, HR = 0.50, 95% CI: 0.25–0.98, P = 0.043; from 2001 to 2010, HR = 0.38, 95% CI: 0.15–0.89, P = 0.026; from 2011 to 2018, HR = 0.21, 95% CI: 0.05–0.86, P = 0.029), and pathological distribution type (PTLBL as reference: HR = 4.71, 95% CI: 1.16–19.22, P = 0.031) were independent risk factors for thymic lymphoma.

### Survival analysis

**Figure 2:**
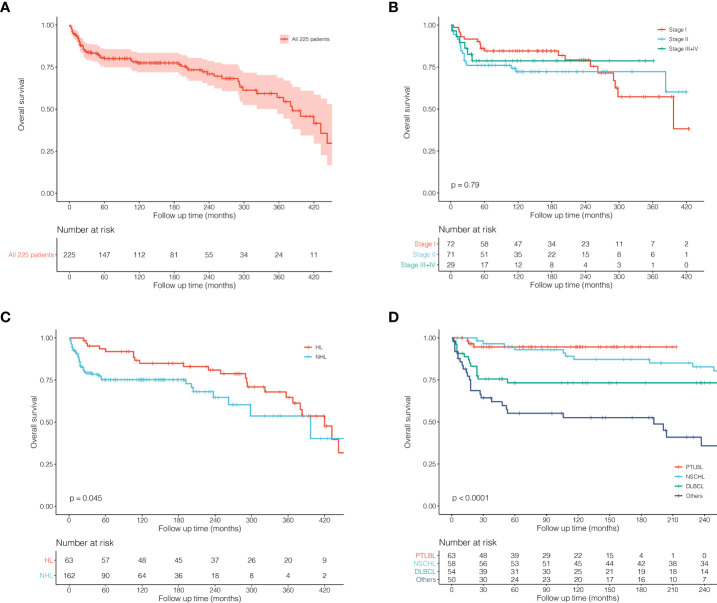
Kaplan–Meier survival curves and log-rank tests for 225 patients with thymic lymphoma from the SEER Research Data with 9 Registries (1975–2018). **(A)** KM survival curves of all 225 patients. **(B)** KM survival curves grouped by Ann Arbor stages. **(C)** KM survival curves grouped by HL and NHL. **(D)** KM survival curves grouped by histological classification.

**Figure 3:**
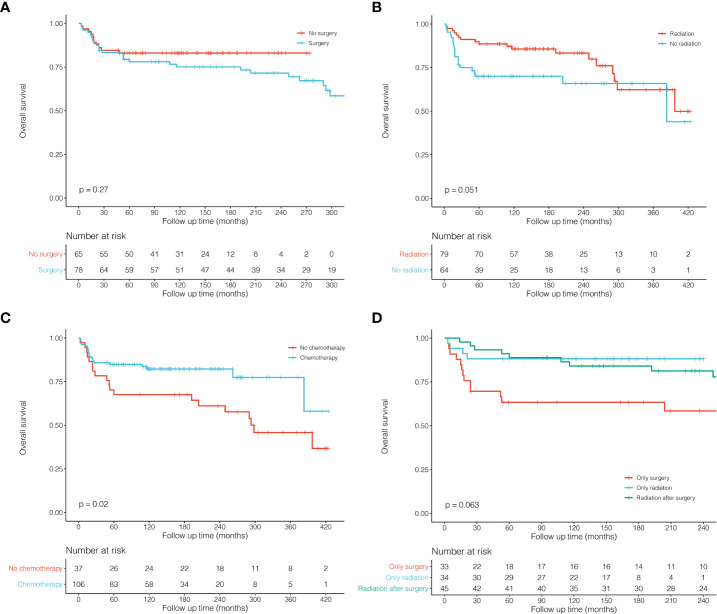
Kaplan–Meier survival curves and log-rank tests for patients with thymic lymphoma (Ann Arbor stage I and II) in different subgroups. **(A)** KM survival curves of patients with thymic lymphoma comparing No surgery and Surgery. **(B)** KM survival curves of patients with thymic lymphoma comparing No radiation and Radiation. **(C)** KM survival curves of patients with thymic lymphoma comparing No chemotherapy and Chemotherapy. **(D)** KM survival curves of patients with thymic lymphoma comparing Only surgery, Only radiation and Radiation after surgery.

Survival analysis of all 225 patients showed a great long-term prognosis (**Figure 2A**). In detail, 5-, 10-, and 20-year OS rates were 80.0%, 77.5%, and 70.9%, respectively. There was no significant difference in long-term survival among different groups of Ann Arbor stages. Stage I had the best prognosis within the first short period (15 years), but there was no statistical difference (**Figure 2B**). The prognosis of HL was significantly better than NHL (P = 0.045; **Figure 2C**). For histological classification, PTLBL had the best prognosis, followed by NSCHL, which was significantly better than DLBCL, while sporadic pathological type had the worst prognosis (P < 0.001; **Figure 2D**). In order to compare the effects of different treatment on the prognosis of thymic lymphoma patients, further survival comparisons were performed on Ann Arbor stage I and II patients (**Figure 3**). As shown in **Figure 3A**, there was no statistically significant difference in prognosis between the surgical group (N = 78) and the non-surgical group (N = 65). The radiotherapy group (N = 79) had better prognosis in the first 25 years than those in the non-radiotherapy group (N = 64), and there was no significant difference for the later period (P = 0.051; **Figure 3B**). Most of the stage I and II patients (N = 106) who received chemotherapy had significantly better prognosis than those who did not (N = 37; P = 0.020, **Figure 3C**). It was shown that patients who received radiotherapy after surgery (N = 45) or radiotherapy alone (N = 34) had better outcome than those who received surgery only (N = 33), although the difference was not significant (P = 0.063; **Figure 3**).

## Discussion

Thymic lymphoma is an extremely rare kind of tumor, and there is lack of large-scale studies or definite guidelines to describe its clinical characteristics, treatment, and prognosis (11–13). The pathologic types of thymic lymphoma mainly included PTLBL, NSCHL, DLBCL, and other sporadic types (4). Timely diagnosis of thymic lymphoma is of great importance to treatment and prognosis (14). Based on pathological characteristics, Andrew et al. reported that the pathological diagnosis accuracy of PTLBL was as high as 92%, and most of the patients could get timely diagnosis according to the characteristics of immunohistochemistry (5). This study firstly used the SEER database to analyze the clinical and prognostic data of the largest cohort of thymic lymphoma and reported the incidence and mortality of the disease.

A total of 233 patients with primary thymus lymphoma were retrieved from the SEER database, of which six cases were dead at diagnosis and two cases lost follow-up. The incidence of thymic lymphoma remained extremely low, about 2.032 per 10 million (95% CI: 1.777–2.312), and it occurred most commonly among middle-age and young adults. Furthermore, a relatively small number of patients were more than 50 years old. It was shown that the number of female patients (127, 56.4%) was slightly more than that of males (98, 43.6%), and the prognosis of females was better than that of males. As for lymphoma types, NHL (162,72.0%) was much more than HL (63,28.0%), and the prognosis of NHL was worse than that of HL. Using univariate and multivariate Cox regression analyses, we found that age and pathologic type were independent prognostic factors. The risk of worse survival outcome increased by 3% for each additional year of age (HR = 1.03, 95% CI: 1.03–1.04, P < 0.001). Secondly, compared with PTLBL, patients with sporadic pathological types had a poor prognosis with a 3.71-fold increased risk (HR = 4.71, 95% CI: 1.16–19.22, P = 0.031).

Overall, patients with thymic lymphoma have a great prognosis with a 5-year OS rate of 80%. The survival curve declined rapidly in the early period with a large number of deaths but tended to be stable after 5 years, indicating the importance of early intervention for thymic lymphoma patients. Besides, considering the rarity of the disease, a limited number of patients we included could cause the much fewer stage III and IV cases. Thus, it might be difficult to accurately reflect the prognostic characteristics of the tumor, and there were no statistical differences observed among survival curves of each stage. HL had a significantly better prognosis than NHL in our study, which was similar to lymphomas of other sites (15). Different pathological types could significantly affect the prognosis of patients with thymic lymphoma (**Figure 2D**). PTLBL showed the best survival outcome: the number of deaths in the early period after diagnosis was much more than that in the later. However, for the long-term outcome, the 5-year survival rate could be up to 90%, and the risk of death in the long-term follow-up was almost zero. It could be inferred that PTLBL might be stable after the early treatment and have a low risk of relapse.

In order to compare the effects of different treatments on prognosis, we conducted survival comparisons among stage I and II patients (**Figure 3** **)**. There was no significant difference between the surgery group and the non-surgery group (P = 0.270), but the radiotherapy group seemed better than that of the non-radiotherapy group, although the difference was not significant (P = 0.051). Next, we performed survival comparisons among patients receiving surgery alone, radiotherapy alone, and postoperative radiotherapy. It can be found that patients receiving radiotherapy alone or postoperative radiotherapy demonstrated better survival than those receiving surgery alone, which suggested that surgical resection alone might have limited effects on survival among thymic lymphoma patients. However, radiotherapy may significantly affect the survival of Ann Arbor stage I and II patients. In addition, most of these patients received chemotherapy, and the chemotherapy group indeed showed much better survival than the non-chemotherapy group. In conclusion, surgical treatment may have limited efficacy on patients with thymic lymphoma, while both radiotherapy and chemotherapy may be more effective and important.

There are also some limitations that should be noted in this study. The analyses we performed were based on the observational and retrospective data from the SEER database. Thus, the data bias and patient heterogeneity could not be totally avoided, but this study can provide some clinical indications. However, considering the rarity of thymic lymphoma, although there were only a limited number of enrolled patients, detailed analyses and comparisons we performed were valuable to provide references for the future studies. This study also showed the largest cohort of thymic lymphoma so far. Besides, clinical information from the SEER database was limited, and a series of important clinical characteristics are unavailable such as immunohistochemical characteristics and details of treatments. Specifically, we generally choose the initial chemotherapy regimen based on the treatment of other primary sites of lymphoma. With the advancement of precision medicine, however, the more effective therapeutics will be developed.

In conclusion, this is the first study to report the incidence and mortality of thymic lymphoma in a largest cohort of thymic lymphoma patients. Univariate and multivariate Cox regression analyses indicated that age and pathological type were independent prognostic factors. In terms of treatment, for Ann Arbor stage I and II patients, surgical treatment has limited effects on OS, while both radiotherapy and chemotherapy may improve survival outcome.

## Data availability statement

The original contributions presented in the study are included in the article/supplementary material. Further inquiries can be directed to the corresponding authors.

## Ethics Statement

This study was reviewed and approved by Ethics Committee of West China Hospital. Written informed consent to participate in this study was provided by the participants’ legal guardian/next of kin.

## Author contributions

Conception and design: LW, ZW, AZ; Provision of study materials or patients: LW, ZW, LH; Collection and assembly of data: LW, LH; Data analysis and interpretation: ZW; Manuscript writing: All authors; Final approval of manuscript: All authors.

## Funding

This study was supported by Post-Doctor Research Project, West China Hospital, Sichuan University (No. 2020HXBH101), “from zero to one” Innovation Research Project of Sichuan University (No. 2022SCUH0025), and China Postdoctoral Science Foundation (No. 2021M692310).

## Conflict of interest

The authors declare that the research was conducted in the absence of any commercial or financial relationships that could be construed as a potential conflict of interest.

## Publisher’s note

All claims expressed in this article are solely those of the authors and do not necessarily represent those of their affiliated organizations, or those of the publisher, the editors and the reviewers. Any product that may be evaluated in this article, or claim that may be made by its manufacturer, is not guaranteed or endorsed by the publisher.
